# Transporter engineering in microbial cell factories: the ins, the outs, and the in-betweens

**DOI:** 10.1016/j.copbio.2020.08.002

**Published:** 2020-12

**Authors:** Steven A van der Hoek, Irina Borodina

**Affiliations:** The Novo Nordisk Foundation Center for Biosustainability, Technical University of Denmark, Kongens Lyngby, Denmark

## Abstract

Engineering the transport of small molecules is an effective approach to improve the performance of microbial cell factories. Transporter engineering can improve the utilization of low-cost alternative substrates, reduce the loss of pathway intermediates, and increase the titer and production rate of the target product. However, transporters are not commonly engineered in strain development programs because the functions of most of the transport proteins are not known. In the recent years, a variety of methods have been developed for identification of transporters for specific substrates and for characterizing transport mechanisms. This review presents recent examples of successful transport engineering for cell factories and discusses the methods for transporter identification and characterization.

## Highlights

• Transporter proteins are essential for substrate uptake, product export, and intercompartmental metabolite exchange.
• Transporter activity of a cell can be engineered to improve the production of metabolites.
• Most of transporters are not functionally characterized.
• Transporter characterization demands specialized techniques, using *Xenopus* oocytes, membrane vesicles, or electrophysiology.

## Graphical abstract

**Figure:**
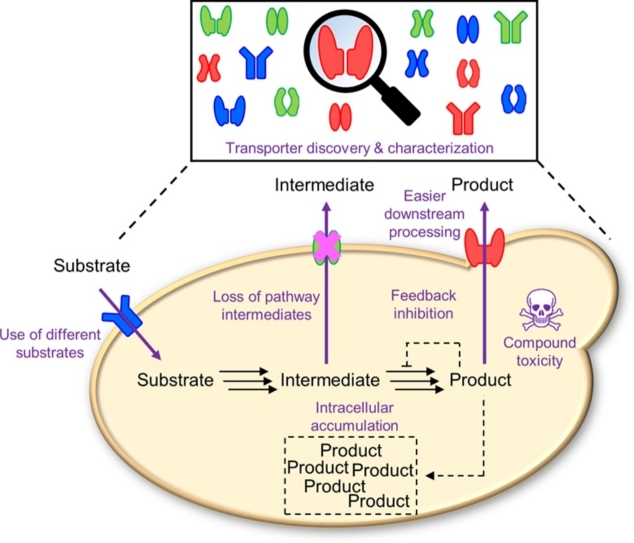

**Current Opinion in Biotechnology** 2020, **66**:186–194

This review comes from a themed issue on **Tissue, cell and pathway engineering**

Edited by **Li Tang**, **Peng Xu** and **Haoran Zhang**

For a complete overview see the Issue and the Editorial

Available online 12th September 2020

**https://doi.org/10.1016/j.copbio.2020.08.002**

0958-1669/© 2020 The Authors. Published by Elsevier Ltd. This is an open access article under the CC BY license (http://creativecommons.org/licenses/by/4.0/).

## Introduction

**Figure 1:**
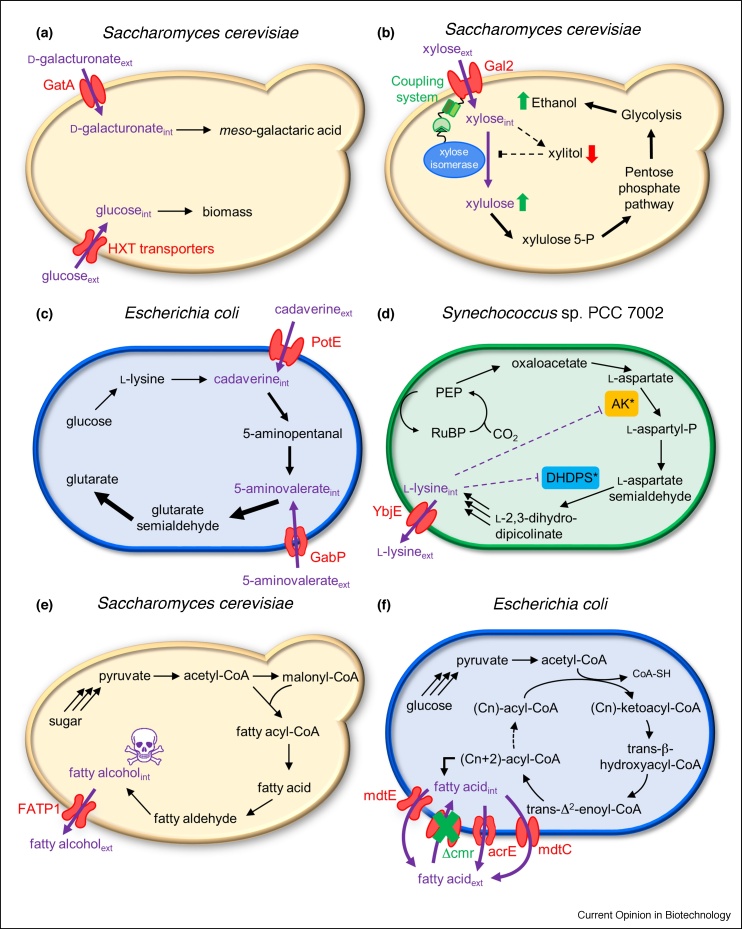
Illustrative case stories of transporter engineering; **(a)** The alternative substrate d-galacturonate is taken up and converted into *meso*-galactaric acid while glucose is used for biomass production; **(b)** The Gal2 transporter is coupled to xylose isomerase, so that when xylose is taken up, it is channeled to the xylose isomerase, decreasing by-product formation and increasing ethanol production; **(c)** The leaked intermediates cadavarine and 5-aminovalerate are re-imported from the medium into the cell by PotE and GabP, respectively, to increase the production of the pathway product glutarate; **(d)** The pathway product lysine is exported by YbjE to reduce feedback inhibition; **(e)** FATP1 exports the fatty alcohol products, reducing toxicity and improving growth; **(f)** Overexpression of *acrE*, *mdtC*, and *mdtE* and deletion of *cmr* simultaneously improves titer limitations by exporting the fatty acid products out of the cell.

Biotechnological production of renewable chemicals and fuels relies on efficient cell factories that convert renewable substrates into products at high titers, rates, and yields [1]. When creating an efficient cell factory, multiple issues must be addressed, and many of them are related to the transport of substrates, biosynthetic intermediates, or products. Transporters that import substrates can increase the substrate uptake rates and hence increase the volumetric productivity, one of the critical determinants of production cost. The uptake of alternative substrates can also be enhanced by transporter engineering, allowing for utilization of mixed complex feedstocks, such as hydrolyzed biomass, organic fractions of municipal solid waste, olive mill waste, and others [2^•^,3^•^] (Figure 1a,b). In the case of multi-step biosynthetic pathways, a common problem is the leakage of the biosynthetic intermediates from the cell. If the leakage of intermediates can be prevented by manipulating transporter activity (Figure 1c), then more carbon will be channeled into the final product, improving the process economics [4^•^].

Transporters that export the final product from the cell can improve product formation by reducing feedback inhibition on the biosynthetic pathway and shifting the reaction equilibrium towards product formation [5]. Export can also enhance the viability of the cells if the product is toxic [6] (Figure 1d,e). Furthermore, exporting the product into the medium simplifies the downstream processing and reduces the overall production cost [7^••^] (Figure 1f).

Despite all the above listed potential benefits of transporter engineering, this approach is not systematically explored in the strain engineering programs, mostly due to the difficulties associated with transporter discovery, characterization, and manipulation [8]. This review summarizes the recent use of transporters in engineering microbial cell factories, as well as methods and tools for transporter discovery and characterization.

## Substrate uptake transporters

Cell factories can be engineered to utilize substrates that they otherwise use only very slowly or not at all. Expansion of the substrate range is essential to enable the use of biomass and waste streams for the production of bulk chemicals and biofuels. However, pretreatment of biomass and waste stream into fermentable sugars releases growth inhibitory compounds. Tolerance against these inhibitors can be achieved by engineering their efflux [9]. Furthermore, substrate uptake is a relevant strategy for the production of some specialty chemicals that require specific precursor molecules; for example, the production of fluorinated compounds requires efficient uptake of fluoride ions from the medium. Here, we discuss several recent examples of successful engineering of substrate uptake in microbial cell factories (Table 1).

**Table 1:** Effect of transporter engineering on cell factory performance

Native organism	Transporter	Cell factory	Transported substrate	Effect of transporter engineering	Reference
**Substrate transporters**					
*S. cerevisiae*	Gal2p	*S. cerevisiae*	Xylose	Coupling to xylose isomerase, increased molar ethanol:xylitol ratio	[3^•^]
*P. chrysogenum*	AraT	*S. cerevisiae*	l-Arabinose	High affinity K_m_ = 0.13 mM, allows growth at 20 g/L glucose	[17]
*E. coli*	XylE	*P. putida*	Xylose	Co-consumption of cellobiose, glucose and xylose when combined with native transporter.	[18]
*A. niger*	GatA	*S. cerevisiae*	d-Galacturonic acid	High transport capacity V_max_ = 12.1 nmol min^−1^ mg^−1^ protein, utilization of d-galacturonic acid from CPW	[2^•^]
*K. lactis*	Lac12	*S. cerevisiae*	Lactose	Enables lactose uptake for the production of 2′-fucosyllactose	[22, 23, 24]
		*Y. lipolytica*			
*E. coli*	Δ*deoD*	*E. coli*	Fluoride	Transporter modifications necessary for *in vivo* fluorination of SAM to 5-fluoro-5-deoxyadenosine	[26^•^]
*R. prowazekii*	samT		SAM		

**Intermediate transporters**					
*E. coli*	GabP	*E. coli*	5-Aminovalerate	72% increase to 2.36 g/L glutarate, Final strain titer 6.21 g/L after deletion of ΔiclR	[4^•^]
	PotE		Cadaverine	11% increase to 3.62 g/L glutarate, final strain titer 6.21 g/L after deletion of ΔiclR	
*E. coli*	FadL	*E. coli*	Palmitate	33.4% increase to 209.4 mg/L ω-hydroxy palmitic acid, combined with 0.1% triton X-100 to achieve 239.1 mg/L ω-hydroxy palmitic acid	[27]
*S. cerevisiae*	Δ*pxa*1	*S. cerevisiae*	Fatty acyl-CoA	14% increase to 254 mg/g CDW	[28]
*E. coli*	ShiA	*E. coli*	3-Dehydroshikimate	∼80% increase to 33 mg/L/OD MA in single cell factory, allows DHS import in coculture system to produce up to 4.7 g/L MA	[29]

**Product transporters**					
*E. coli*	YbjE	*Synechococcus* sp. PCC 7002	Lysine	Rescues growth and allows for lysine export, final strain can produce up to 3.11 mM lysine	[5]
*S. cerevisiae*	FATP1	*S. cerevisiae*	1-Alkenes	37% increase to 35.3 mg/L 1-alkenes	[30]
*S. cerevisiae*	FATP1	*S. cerevisiae*	Fatty alcohol	77% increase to 240 mg/L fatty alcohols	[6]
*S. cerevisiae*	Qdr3	*S. cerevisiae*	Muconic acid	64% increase to 0.41 g/L muconic acid, confers tolerance to glutaric, adipic, muconic, glutaconic acid	[31]
*A. niger*	CexA	*A. niger*	Citrate	354% increase to 109 g/L citrate	[33^••^]
*E. coli*	AcrE	*E. coli*	Fatty acids	∼135% increase to 1781 mg/L fatty acids	[7^••^]
	MdtC				
	MdtE				
	Δ*cmr*				

The transport of xylose and arabinose, two most abundant pentoses in biomass hydrolyzates, has been studied extensively in yeast *Saccharomyces cerevisiae* for the production of second-generation bioethanol [10]. *S. cerevisiae*, like many other microbes, prefers glucose as the carbon source and has a glucose catabolite repression mechanism, where the utilization of other sugars is delayed until all glucose has been consumed. This gives longer fermentation times and lowers the volumetric productivity [11,12]. An interested reader is referred to an extensive review on improving sugar uptake in *S. cerevisiae* [13]. A novel strategy for improving xylose uptake was described by Thomik *et al.*, who coupled the Gal2p transporter to xylose isomerase from *Clostridium phytofermentans* in *S. cerevisiae*. The coupling was achieved by tagging the transporter and the xylose isomerase with protein–protein interaction domains. The rationale was to direct the imported xylose to the catabolic enzyme and avoid the formation of by-product xylitol by native aldose reductases. Indeed, the coupling increased the molar ethanol-to-xylitol ratio from 2 to 5.5 [3^•^].

Constructing *S. cerevisiae* strains capable of utilizing arabinose in the presence of glucose is problematic, as glucose, even at low concentration, strongly inhibits the uptake of known arabinose transporters [14, 15, 16]. Therefore, the transcriptome of *Penicillium chrysogenum* was analyzed in arabinose-limited cultivation to identify arabinose-specific transporters. Sixteen candidate genes were found with at least threefold higher transcript levels compared to glucose-limited and ethanol-limited conditions, and after homology search, the top five candidate genes were screened for arabinose uptake in *S. cerevisiae*. The newly identified *Pc*AraT had a high affinity for arabinose (K_m_ = 0.13 mM versus 335 mM for Gal2p) and did not transport glucose or xylose. A strain that had a deletion of *GAL2* and expressed *Pc*AraT grew at 0.099 h^−1^ on 20 g/L arabinose and at 0.057 h^−1^ on a mix of 20 g/L each of arabinose and glucose [17]. Thus *Pc*AraT was much less inhibited by glucose than other known arabinose transporters. For example, LAT-1 from *Neurospora crassa* and MtLAT-1 from *Myceliophthora thermophilia* had their transport capacity inhibited by over 95% when tested in medium containing 15 g/L arabinose and 18 g/L glucose [16].

*Pseudomonas putida* was also recently engineered for the uptake of cellobiose and pentoses. *P. putida* is an attractive host for the utilization of biomass hydrolyzates and various waste streams due to its high natural tolerance to toxic metabolites often present in these feedstocks. The uptake of xylose was enabled by the expression of *Escherichia coli* proton-coupled symporter XylE. To prevent the conversion of xylose into a side product d-xylonate, the authors inactivated the enzyme that performs the reaction — quinone-dependent glucose dehydrogenase. Interestingly, this enzyme is also required for glucose utilization via gluconate, and after its deletion, the cell apparently took up glucose via the ABC glucose transporter instead. The same transporter also imported cellobiose. The engineered strain co-consumed 2 g/L of cellobiose, xylose, and glucose each within 28 hours [18].

In some cases, a particular compound is added to the process not as the carbon or energy source, but for direct incorporation into the final product. Pulpy wastes from the juicing of cash crops such as apples, grapes, and agave contain between 20–40% of the dry weight in pectin [19], a polysaccharide made of over 70% of the monomer d-galacturonic acid [20]. In Protzko *et al*. [2^•^], baker’s yeast was engineered to produce *meso*-galacteric acid from d-galacturonic acid in citrus peel waste (CPW) hydrolysate. A uronate dehydrogenase coding gene from *Agrobacterium tumefaciens* was integrated into the genome of *S. cerevisiae* to enable the conversion of d-galacturonic acid into *meso*-galactaric acid. As *S. cerevisiae* grew in a biphasic manner on a mixture of glucose and d-galacturonic acid, the transport of d-galacturonic acid had to be engineered. A high-affinity transporter for d-galacturonic acid (K_m_ = 1 μM) was previously identified in *N. crassa.* However, it had a low transport capacity (V_max_ = 0.256 nmol min^−1^ mg^−1^ protein) [21]. Therefore, the authors screened 13 homologs from *Aspergillus niger* and found a new glucose-insensitive d-galacturonic acid transporter GatA with a lower affinity (K_m_ = 340 μM), but a 47-fold higher transport capacity (V_max_ = 12.1 nmol min^−1^ mg^−1^ protein). Baker’s yeast equipped with this transporter produced 0.52 mol *meso*-galactaric acid mol^−1^ d-galacturonic acid in 80 hours from CPW hydrolysate supplemented with 200 g/L glucose, compared to 0.13 mol *meso*-galactaric acid mol^−1^ d-galacturonic acid produced by a strain without the transporter [2^•^].

Another example of engineering the uptake of unusual substrates for bioconversion is the production of human milk oligosaccharides (HMO) by yeast. HMOs are important prebiotics present in mother’s milk; they are produced recombinantly for addition to baby milk formulas. 2′-fucosyllactose is the most abundant HMO and can be produced from glucose and lactose. As *S. cerevisiae* does not take up lactose naturally, the production of 2′-fucosyllactose was enabled by the expression of the Lac12 transporter of *Kluyveromyces lactis*. Yu *et al.* and Liu *et al.* reported titers of ∼0.5 g/L in *S. cerevisiae* [22,23], while Hollands *et al.* further optimized the expression of the pathway enzymes to obtain 2′-fucosyllactose titer of 15 g/L in *S. cerevisiae* and 24 g/L in *Yarrowia lipolytica* [24].

Fluorination is an essential chemical reaction widely used to produce materials and medicine [25], but it has no biotechnological alternative yet. Recently, *in vivo* fluorination was achieved in *E. coli* by accumulating the reaction substrates fluoride and *S*-adenosylmethionine (SAM), which are then used by a fluorinase from *Streptomyces* sp. *MA37* to produce 5-fluoro-5-deoxyadenosine. SAM uptake was increased by incorporating the *samT* transporter from *Rickettsia prowazekii* RP076, and the accumulation of the substrate fluoride was accomplished by deleting the native *crcB* gene responsible for fluoride export. Only then was the strain capable of producing 5-fluoro-5-deoxyadenosine from fluoride and SAM, as strains carrying the fluorinase gene and only one of the two transporter modifications did not produce 5-fluoro-5-deoxyadenosine [26^•^].

## Transport of pathway intermediates

There are primarily two strategies that have been used so far to combat the loss of pathway intermediates: re-uptake of the leaked intermediates and preventing the leakage of the intermediates altogether (Table 1). When *E. coli* was used for the production of glutarate through the native lysine pathway, biosynthetic intermediates 5-aminovalerate and cadaverine were secreted at 1.19 g/L and 1.66 g/L, respectively. Overexpression of uptake transporters GabP and PotE improved the re-uptake of these intermediates and enhanced glutarate production by 72% and 11%, respectively [4^•^]. In another example, *E. coli* was used to produce ω-hydroxy palmitic acid from glucose, but at the same time, palmitic acid was secreted at ∼100 mg/L. This caused the loss of the energetically costly intermediate and also created a problem with purification, as ω-hydroxy palmitic and palmitic acids are difficult to separate. Therefore, the native fatty acid transporter FadL was overexpressed, reducing the amount of secreted palmitic acid by ∼70% [27]. During the production of lipids in *S. cerevisiae*, part of the acyl-CoAs precursors are transported into peroxisomes by the peroxisomal fatty acyl-CoA transporter Pxa1p and are then degraded by β-oxidation. The deletion of *PXA1* improved lipid content in the cells by 14% to 254 mg g^−1^ CDW [28].

Lastly, intermediate transporters have also been used in co-culture systems that aim to separate and optimize biosynthetic pathways. In Zhang *et al*. [29], the production of *cis,cis*-muconic acid (MA) in *E. coli* was improved ∼80% to 33 mg L^−1^ OD^−1^ by expressing the shikimate transporter ShiA of *E. coli* to take up the intermediate 3-dehydroshikimic acid (DHS) that accumulated in the media. However, DHS accumulation was still at 46 mg L^−1^ OD^−1^. Therefore, the authors used the *shiA* gene to separate the production of the intermediate DHS in one *E. coli* strain, and implement the uptake and conversion of DHS to MA in another *E. coli* strain. By using a co-culture system with these two strains and making use of the ShiA transporter, the MA production was increased to ∼590 mg L^−1^ OD^−1^, at a yield 0.35 g g^−1^ sugar, ca. half of the theoretical maximum yield [29].

## Export of products

If the product is retained within the cell, it may inhibit the upstream biosynthetic enzymes, cause cellular toxicity, or slow down the reaction rates of the biosynthesis pathway as the reactions approach chemical equilibrium. Therefore improving the product export is an attractive strain engineering strategy (Table 1). In cyanobacterium *Synechococcus* sp. PCC 7002, lysine inhibits two enzymes in its own biosynthesis, dihydrodipicolinate synthase and aspartate kinase. Expression of a feedback-insensitive version of dihydrodipicolinate synthase led to a growth defect, and the resulting strain could not be further engineered. In alternative strategy, the lysine exporter YbjE from *E. coli* was expressed and this increased the extracellular concentration of lysine from <0.01 μM to ∼0.13 μM [5]. In *S. cerevisiae*, the human fatty acid transporter FATP1 was used to improve the production of both 1-alkenes and fatty alcohols, as these products are similar to the fatty acid substrate of FATP1. Expression of FATP1 improved the extracellular and total 1-alkenes titer by 40% and 37%, respectively [30]. For fatty alcohols, the expression of FATP1 increased the extracellular fatty alcohol titer from 15 mg/L to 70 mg/L, with the total fatty alcohol production increasing by 77% to 240 mg/L. Furthermore, it improved the growth of the strain by 2.5-fold as fatty alcohols are toxic for baker’s yeast [6]. Overexpression of multidrug resistance transporter Qdr3p in *S. cerevisiae* confers tolerance to a wide range of dicarboxylic acids, such as glutarate, adipate, pimelate, muconate, and glutaconate, but not malate, succinate or malonate. Overexpressing this transporter in muconate-producing strain improved the muconate titer by 64% to 0.41 g/L [31]. The transporter of citric acid CexA in *A. niger* was recently identified by characterizing ten homologs of *Ustilago maydis* itaconic transporter Itp1 [32]. Integrating *cexA* under the control of an inducible promoter improved the citrate production of *A. niger* by 354% to 109 g/L [33^••^]. In a systematic approach to improve the fatty acid production in *E. coli*, seventeen transporter genes were engineered. The overexpression of *mdtE, mdtC,* and *acrE,* as well as the deletion of *cmr,* all improved the fatty acid export. When these four transporter modifications were combined, the intracellular fatty acid pool decreased from approximately 105 mg/L to 25 mg/L, while the extracellular fatty acid titer increased from ∼600 mg/L to ∼1750 mg/L [7^••^].

## Transporter discovery and characterization

**Figure 2:**
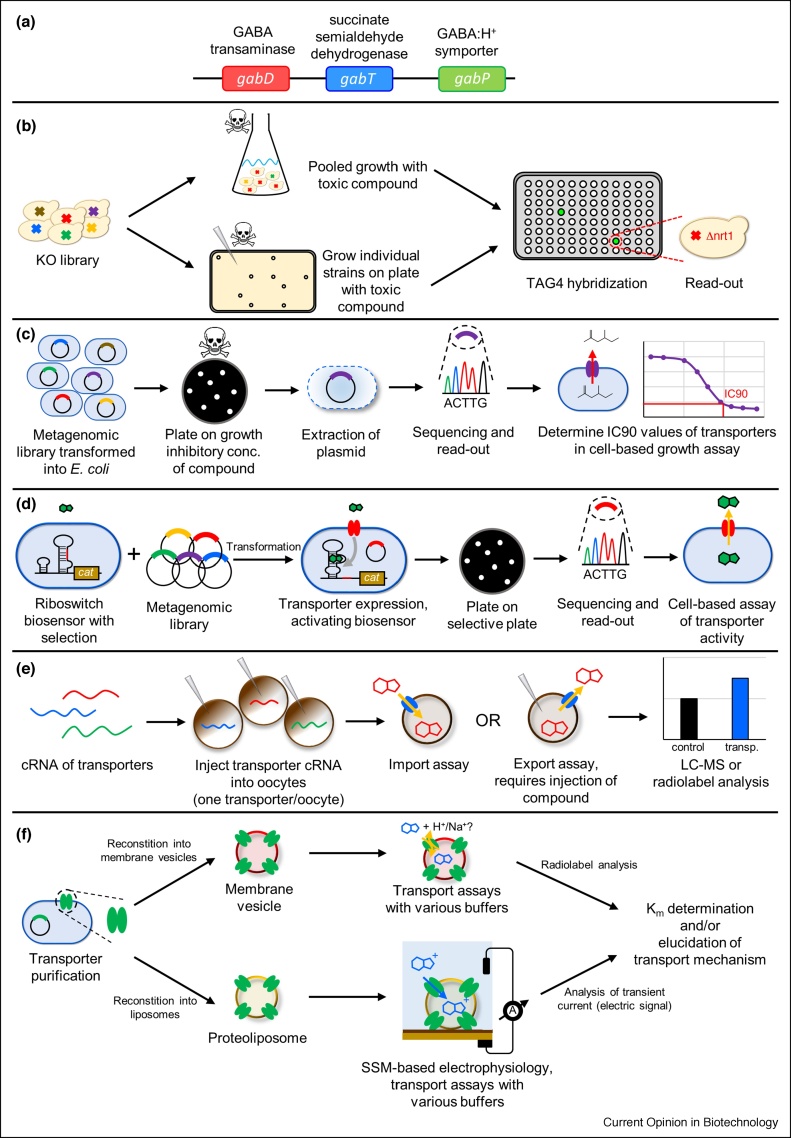
Transporter discovery and characterization methods; **(a)** Function prediction based on transporter localization in a gene cluster within the genome; **(b)** Screening a knock-out library by growth on toxic concentrations of compounds identifies importers; **(c)** Screening of a metagenomics library by plating on growth inhibitory concentrations of a compound of interest identifies new exporters; **(d)** Biosensor-based screens of a metagenomics library identify new transporters; **(e)** Expression of transporters in *Xenopus laevis* oocytes combined with import/export assays is used for the identification and characterization of transporters; **(f)** Transporter characterization using membrane vesicles or by SSM-based electrophysiology under different assay conditions.

The engineering of metabolite transport is difficult due to the limited information about transporter specificity. As illustrated in the literature examples above, most of the transporter engineering studies included transporter discovery work as well. The methods for the discovery and characterization of transporters are summarized in Figure 2.

The most straightforward method is to search for homologs of known transporters to find transporters for similar substrates [33^••^] or transporters with improved kinetics [2^•^]. In bacteria, transporters can sometimes be found in their biosynthetic gene clusters, as is the case for the minimycin transporter MinT of *Streptomyces hygroscopicus* JCM 712 [34] and *gabP* in the *gabDTP* operon of *E. coli* [4^•^] (Figure 2a). AntiSMASH is a powerful tool to identify gene clusters, and it also annotates transporters within the gene clusters [35]. The predicted heterologous transporter candidates are typically expressed in the cell factory, and the effect on product formation is evaluated. Alternatively, for identifying the native transporters, the candidate genes are deleted, and the effect is evaluated. The latter approach is, however, often unsuccessful due to the high redundancy of transporters. For example, in search of the lactate transporter in *S. cerevisiae*, 25 transporter genes were deleted, but the specific lactate production rate did not change [36].

The key disadvantage of the previously mentioned methods is that they are low-throughput. The following examples will thus focus on high-throughput methods for transporter characterization. If the compound of interest exhibits toxicity, a tolerance screen can be set up for screening a large library of strains with deletions or (over)expressions of transporters. A previously constructed barcoded deletion library of *S. cerevisiae* [37] was subjected to a growth challenge at the IC_90_ of the toxic compound diphenyleneiodonium chloride (DPI). Enrichment of *Δnrt1* mutant was found by barcode sequencing, suggesting that Nrt1p is the importer of DPI. Importers of 26 toxic drugs were found in *S. cerevisiae* using this method [38] (Figure 2b). For finding efficient exporters for lysine, a metagenomics library was constructed from cow fecal samples and expressed in *E. coli*. The strains were selected by plating on inhibitory concentrations of lysine, and a new lysine exporter *mglE* was found. Expressing this exporter in an industrial *Corynebacterium glutamicum* strain improved the lysine production by 12% [39] (Figure 2c).

In Ref. [40], high-throughput transporter screening was mediated by a riboswitch-based biosensor that enabled the expression of chloramphenicol and spectinomycin resistance upon activation by thiamine pyrophosphate. With this biosensor system, a metagenomics library prepared from soil and fecal samples was screened on plates containing chloramphenicol and spectinomycin. Subsequent sequencing of surviving colonies led to the discovery of the PnuT thiamine transporter family [40] (Figure 2d).

The assays described above rely on toxicity of the target compound or on availability of a biosensor for selection. As an alternative high-throughput technique, transporter libraries can be systematically screened in *Xenopus laevis* oocytes against compounds of interest. The immature eggs (oocytes) of the South African clawed frog *X. laevis* oocytes can reach 1 mm in diameter and are therefore suitable for micromanipulation. When injected with cRNA encoding the transporter of interest, they will readily express the transporter protein in their membranes. The oocytes have been successfully applied for expressing multiple bacterial, plant, and mammalian transporters. The native transport activity background of oocytes is low [41]. The oocytes expressing the transporter of interest can be used for import assays, when they are placed in a solution with the metabolite of interest and the concentration of metabolite is then measured in the solution and inside the oocytes. They can also be used for export assays, where a compound(s) is injected into the oocyte, and, after some incubation in a buffer, the intracellular and extracellular concentrations of the compound(s) are measured. On the downside, the method is rather resource intensive and costly. However, the method provides reliable information on transporter specificity and sometimes also directionality, and is therefore still widely used. A large library of transporters is currently only available for *Arabidopsis thaliana* [42^•^]. It has been used to identify a new glucose transporter AtSTP13 and two glucosinolate transporters, GTR1 and GTR2 [42^•^,^43^] (Figure 2e).

After the discovery of a new transporter, the characterization becomes important as the transporter characteristics are vital parameters for cell factory engineering. The Mae1 transporter from *Schizosaccharomyces pombe* was shown to have a significantly better export activity for fumarate, malate, and succinate, compared to other dicarboxylate transporters in *Xenopus* oocyte experiments (Figure 2e). Furthermore, SpMae1 increased the production of malate in yeast *S. cerevisiae* without imposing a growth defect, as was the case with the other transporters. Organic acid transporters typically require proton, sodium, or ATP motive force for exporting acids from the cells, effectively draining part of the cellular energy and decreasing the growth. By studying protein motifs and conserved amino acid residues of SpMae1, the authors showed that SpMae1 belongs to the voltage-dependent slow activating (S-type) anion channel 1 (SLAC1) family and therefore does not require energy expenditure for acid transport [44].

*In vitro* assays are the only methods that allow extensive manipulation of internal and external buffer compositions and are thus necessary to fully characterize transporters. In one variation of the method, radiolabeled substrates are used in conjunction with membrane vesicles. The C_4_-dicarboxylate transporter DctA of *Bacillus subtilis* was overexpressed in *Lactococcus lactis,* and the cells were disrupted to obtain DctA-containing membranes. These were subsequently mixed with lipids from *E. coli* to generate fused membrane vesicles. Transport assays using radiolabeled substrates (succinate, fumarate, oxaloacetate, and malate) and a variety of buffers with different pH values were then used to determine that DctA is a dicarboxylate-proton symporter [45] (Figure 2f). Alternatively, transporter characterization can be performed using solid-supported membrane-based (SSM-based) electrophysiology. In SSM-based electrophysiology, membrane vesicles or proteoliposomes adhere to a membrane supported by a gold layer. This set-up ensures the capacitive coupling of the proteoliposome with the gold layer, allowing the user to measure electrogenic transport [46]. Electrogenic transport is any transport event that transports a net charge (e.g. an uncharged substrate with a proton or ion co-substrate) over the membrane. Using this technique, the transport mechanisms of the lactose permease LacY, the fucose permease FucP, and the xylose transporter XylE, all from *E. coli*, were further investigated. By varying the substrate concentrations and pH values of the buffers used during the assay, the authors measured in detail the K_m_ values, the pH dependency, and acid and alkaline pH inhibition points of the three proton symporters [47] (Figure 2f).

## Conclusions and perspectives

The recent work within transporter engineering of microbial cell factories clearly shows the positive and significant effects transporters have on the bioproduction of value-added chemicals (Table 1). Nevertheless, for metabolic engineers to widely adapt transporter engineering in microbial cell factories, further knowledge on a vast range of transporters needs to be accumulated.

However, current methods for the identification of transporters have constraints and limitations, which range from throughput issues to the need for a biosensor or a toxicity profile. Similarly, the characterization of transporters is very laborious and tedious due to the need to optimize the purification of the transporter protein or the hands-on work required in *Xenopus* oocyte experiments. Furthermore, even when a transporter is available, unexpected problems may arise when applying it to a microbial cell factory, such as expression issues, improper localization, or growth inhibition.

Therefore, it would be a significant advancement for the field to develop a low-cost, high-throughput, automated method for the identification or characterization of transporters that can work with most transporters and substrates. Simultaneously, tools have to be developed that allow the proper expression and localization of transporters so that they can be introduced to virtually any microbial cell factory. The RESOLUTE consortium was recently initiated to generate data, tools, and strains for human Solute carrier (SLC) transporters [48]. While heterologous membrane proteins do not always express well [49], the RESOLUTE consortium will surely enable and inspire microbial transporter studies. At the same time, ongoing efforts within the metabolic engineering community will generate data on compounds produced in cell factories, and develop tools to implement transporters into cell factories effectively. In conclusion, the engineering of transport within cell factories is a scientifically challenging but rewarding field that will have a significant impact on future metabolic engineering efforts.

## Conflict of interest statement

Nothing declared.

## References and recommended reading

Papers of particular interest, published within the period of review, have been highlighted as:

• of special interest
•• of outstanding interest

## CRediT authorship contribution statement

**Steven A van der Hoek:** Conceptualization, Visualization, Writing - original draft. **Irina Borodina:** Conceptualization, Writing - review & editing, Supervision, Funding acquisition.
